# Population Screening for Chronic Q-Fever Seven Years after a Major Outbreak

**DOI:** 10.1371/journal.pone.0131777

**Published:** 2015-07-01

**Authors:** Gabriëlla Morroy, Wim van der Hoek, Jelle Albers, Roel A. Coutinho, Chantal P. Bleeker-Rovers, Peter M. Schneeberger

**Affiliations:** 1 Department of Infectious Disease Control, Municipal Health Service Hart voor Brabant, 's-Hertogenbosch, the Netherlands; 2 Department of Primary and Community Care, Academic Collaborative Centre AMPHI, Radboud University Medical Center, Nijmegen, the Netherlands; 3 Epidemiology and Surveillance Unit, Centre for Infectious Disease Control, National Institute for Public Health and the Environment (RIVM), Bilthoven, the Netherlands; 4 Julius Center for Health Sciences and Primary Care, University Medical Center, Utrecht, the Netherlands; 5 Department of Internal Medicine, Division of Infectious Diseases, Radboud Expertise Center for Q-fever, Radboud university medical center, Nijmegen, the Netherlands; 6 Department of Medical Microbiology, Jeroen Bosch Hospital, 's-Hertogenbosch, the Netherlands; Texas A&M Health Science Center, UNITED STATES

## Abstract

**Introduction:**

From 2007 through 2010, the Netherlands experienced a large Q-fever epidemic, with 4,107 notifications. The most serious complication of Q-fever is chronic Q-fever.

**Method:**

In 2014, we contacted all 2,161 adult inhabitants of the first village in the Netherlands affected by the Q-fever epidemic and offered to test for antibodies against *Coxiella burnetii* using immunofluorescence assay (IFA) to screen for chronic infections and assess whether large-scale population screening elsewhere is warranted.

**Results:**

Of the 1,517 participants, 33.8% were IFA-positive. Six IFA-positive participants had an IgG phase I titer ≥1:512. Two of these six participants were previously diagnosed with chronic Q-fever. Chronic infection was diagnosed in one of the other four participants after clinical examination.

**Conclusions:**

Seven years after the initial outbreak, seroprevalence remains high, but the yield of screening the general population for chronic Q-fever is low. A policy of screening known high-risk groups for chronic Q-fever in outbreak areas directly following an outbreak might be more efficient than population screening. A cost-effectiveness analysis should also be performed before initiating a population screening program for chronic Q-fever.

## Introduction

Q-fever is a zoonotic disease caused by the bacterial pathogen *Coxiella burnetii (C*. *burnetii)*[1,2]. From 2007 through 2010, the Netherlands experienced a large Q-fever epidemic, with 4,107 notifications [3]. Prior to 2007, the seroprevalence of Q-fever antibodies among the Dutch general population was 2.4% [3]. In 2007, a sample of the inhabitants of Herpen—the first Dutch village affected by the Q-fever outbreak—had a seroprevalence rate of 25.1% [4].

The most serious complication of Q-fever is chronic Q-fever. Chronic Q-fever develops in 1.5–2% of Q-fever infections and can be detected months—or even years—after the initial infection, which was either symptomatic or asymptomatic [5]. The risk factors for chronic Q-fever include pre-existing cardiac valvulopathy, vascular graft, aneurysm, and immunosuppression [5]. Chronic *C*. *burnetii* infection can lead to endocarditis, an infected aneurysm or vascular graft, causing high morbidity and mortality even if optimal treatment is received [1,6]. Because chronic Q-fever is not classified as a notifiable disease, precise numbers are not available; however, up to May 2012, 284 patients were voluntarily registered into a database as part of a research project run by the University Medical Center Utrecht [7]. For early detection of chronic Q-fever, patients should have at least one serological examination within one year following the acute infection [8]. The serological follow-up screening of acute Q-fever patients varies widely among regions, ranging from 25% to 95% [9].

General practitioners (GPs), inhabitants of regions with a high Q-fever incidence and the Dutch national Q-fever patient organization, speculated that the number of chronic Q-fever cases of the 2007–2010 epidemic was underestimated. Chronic Q-fever was incidentally diagnosed years after asymptomatic infection but the extent was never quantified. Therefore, seven years after the initial outbreak in the Netherlands, we measured the serological *C*. *burnetii* status of inhabitants of the high incidence village Herpen in order to identify chronic Q-fever infections and assess whether large-scale population screening elsewhere is warranted.

## Methods

The Municipal Health Service (MHS) “*GGD Hart voor Brabant*” performed this study as part of the larger Q Herpen II project. The Medical Ethics Review Committee of the University Medical Center Utrecht approved the study (protocol 13-367/D Q Herpen II).

For this cross-sectional population study, all adult inhabitants (≥18 years of age) of the village of Herpen (Dutch postal code 5373) were invited to participate. The municipal administration provided demographic data for these 2,161 inhabitants. In January 2014, all inhabitants were sent an information package by post, including information regarding the study, a request to participate, a questionnaire, an informed consent form, and a laboratory form for venipuncture.

The questionnaire included questions regarding demographics, smoking history, risk factors associated with chronic Q-fever, history of Q-fever infection and vaccination. Answers to questions about general health status, initial symptoms, chronic medical conditions, and medication use are currently being analyzed in other sub-studies.

During five days in February and one day in March 2014, participants provided their written informed consent to participate in this study with the questionnaire. Informed consent forms and questionnaires were checked for missing information and errors by medical staff and the participant, followed by a venipuncture.

### Diagnosis

Antibodies against *C*. *burnetii* were measured using immunofluorescence assay (IFA) see Supplementary Information (SI) S1 Text, and an IgG phase I or II titer ≥1:64 was interpreted as IFA-positive.

The Dutch Q-fever Consensus Group [10] considers an IgG phase I titer ≥1:1024 an indication of possible chronic Q-fever; for a definitive diagnosis, a comprehensive medical examination is required. In our study, given the lack of an initial clinical examination, the serological cut-off value was set one dilution lower (at IgG phase I 1:512) in order to maximize sensitivity.

Participants with an IgG phase I titer ≥1:512 were tested further using the Q-fever polymerase chain reaction test (PCR) S1 Text, and referred to the Q-fever clinic at Radboud university medical center (Radboudumc) for clinical examination, including echocardiography and positron emission tomography-computed tomography (PET-CT) [10], when deemed necessary.

The IFA results were reported to the participants and their GP together with a medical recommendation.

### Previous infections

The IFA test results of this study in 2014 were compared with those obtained in 2007. The 2007 and 2014 studies were performed in the same village (Herpen) and used the same laboratory tests and cut-off values [4]. Data regarding previous Q-fever infections and notifications were obtained from the MHS.

### Data analysis

Questionnaires were digitally scanned and analyzed using SPSS 21.0. The age and gender of the non-responders were obtained from the municipal administration data. Proportions were compared using the chi-square test. Differences with p<0.05 were considered to be significant (two-tailed analysis). The independent sample t-test was used to calculate means.

## Results

### The study population

Both a blood sample and a completed questionnaire were provided by 70.2% (N = 1,517/2,161) of the adult inhabitants of Herpen, the Netherlands. The characteristics of the participants are summarized in Table 1.

**Table 1:** Characteristics of the study participants.

	All		IFA-Positive		IFA-negative		
	N = 1,517	*(100%)*	N = 513	*(33*.*8%)*	N = 1,004	*(66*.*2%)*	p-value
**Mean age, years** ^**1**^	51.9	(SD 16.5)	51.6	(SD 15.7)	52.1	(SD 16.9)	0.58
**Gender** ^**2**^							0.70
Male	752	*(49*.*6)*	258	*(50*.*3)*	494	*(50*.*8)*	
Female	765	*(50*.*4)*	255	*(49*.*7)*	510	*(49*.*2)*	
**Smoking** ^**2**^							0.02
Current	276	*(18*.*3)*	110	*(21*.*4)*	166	*(16*.*6)*	
Former	570	*(37*.*7)*	194	*(37*.*8)*	376	*(37*.*6)*	
Never	666	*(44*.*0)*	209	*(40*.*8)*	457	*(45*.*8)*	
**Education level** ^**2**^ ^,^ ^**3**^							0.25
Low	825	*(55*.*2)*	290	*(57*.*3)*	535	*(54*.*1)*	
Average	425	*(28*.*4)*	149	*(29*.*4)*	276	*(27*.*9)*	
High	245	*(16*.*4)*	67	*(13*.*2)*	178	*(18*.*0)*	
**All cardiovascular risk factors** ^**4**^	93		22		71		
One or more cardiovascular risk factors	69		16		53		
Aneurysm	19	*(20*.*4)*	8	*(36*.*4)*	11	*(15*.*5)*	
Aortic bifurcation prosthesis	2	*(2*.*2)*	0	*(0*.*0)*	2	*(2*.*8)*	
Stent graft	40	*(43*.*0)*	6	*(27*.*3)*	34	*(47*.*9)*	
Tube graft	1	*(1*.*1)*	1	*(4*.*5)*	0	*(0*.*0)*	
Bypass	20	*(21*.*5)*	3	*(13*.*6)*	17	*(23*.*9)*	
Heart valve surgery	11	*(11*.*8)*	4	*(18*.*2)*	7	*(9*.*9)*	

^1^Independent sample *t*-test

^2^Pearson’s chi-square test. For the purpose of the analysis current smoking was compared with past and never smoked and for education level low was compared with average combined with high.

^3^Education level: low, ranging from no education to vocational training; average, ranging from secondary vocational education to preparatory academic training and high, higher professional and/or university education.

^4^The p-value was not calculated for this heterogeneous group

Participants and non-participants were similar with respect to age (p = 0.31) and gender (p = 0.35). The mean age of the participants and non-participants was 51.9 years (SD: 16.5 years) and 51.2 years (SD: 21.9 years), respectively. More participants with Q-fever (N = 51/1,517) were notified by the MHS compared to non-participants with Q-fever (N = 2/644; p<0.01).

#### Prevalence of antibodies against *C*. *burnetii*

Of the 1,517 participants, 513 (33.8%) tested positive for antibodies against *C*. *burnetii* (i.e., were IFA-positive; for titers see S1 Table). Three of the 513 IFA-positive participants became seropositive after receiving a Q-fever vaccination in 2011; two other vaccinated participants were seronegative in 2014. The IFA-positive and IFA-negative participants were similar with respect to age, gender and education level. A risk factor for being IFA-positive was current smoking (OR 1.4; 95% CI: 1.05–1.80; p = 0.02) versus former smoker and never smoked.

Of the 513 IFA-positive participants, six (1.2%) had an IgG I ≥1:512, Table 2 and a negative Q-fever PCR test. Two of these six participants were diagnosed previously with chronic Q-fever. The remaining four (two with an IgG phase I 1: 512) were referred for a comprehensive clinical examination; three of these participants had no evidence of a chronic *C*. *burnetii* infection and one participant-a male over the age of 65, with an increased erythrocyte sedimentation rate and a history of renal insufficiency, and diabetes mellitus type 2- was diagnosed with chronic Q-fever. This participant presented with a cardiac murmur, and although transesophageal echocardiography revealed no signs of endocarditis, he was placed on a treatment regimen consisting of doxycycline (200 mg qd) and hydroxychloroquine (200 mg tid).

**Table 2:** Summary of the six participants with an IgG I titre ≥1:512 and Q-fever status after clinical examination.

Patient	IgG phase I	IgG phase II	Gender	Age*	Initial symptoms	Year diagnosis Q-fever	Underlying disease	Chronic Q-fever diagnosed
1	1:512	1:4096	female	<65	yes	2007	None	no
2	1:512	1:4096	male	<65	no	2014	None	no
3	1:1024	1:1024	female	≥65	no	2014	Diabetes mellitus type II	no
4	1:1024	1:2048	male	≥65	yes	2008	Aneurysm + stent	yes, 2008
5	1:1024	1:2048	male	≥65	yes	2010	Heart valve surgery	yes, 2011
6	1:1024	1:4096	male	≥65	no	2014	Diabetes mellitus type II	yes, 2014
							Impaired renal function	

*The age is not shown as the exact age of the participant as this could compromise the privacy of the individual. Participants 1, 2,3, and 6 were due this study referred for a comprehensive clinical examination to exclude chronic Q-fever. Participants number 4, 5 and 6 were diagnosed with chronic Q-fever; number 4 after the development of an aneurysm, number 5 during screening before vaccination of high risk groups and, number 6 as a consequence of screening during the current study.

Of the 69 individuals with known cardiovascular risk factors, 16 (23.2%) were IFA-positive Table 1; three of the 16 participants previously received the Q-fever vaccine, and the other 13 were exposed to Q-fever naturally. Of these 13 participants two (15.4%) developed a chronic infection. These were the two participants (out of the six with an IgG I ≥1:512) previously diagnosed with chronic Q-fever.

#### Comparison of the 2014 IFA test results with previous Q-fever tests

In 2007, 25.3% (111/443) of sampled adult inhabitants in Herpen were IFA-positive. We compared results from 287 individuals who participated in both studies and gave their consent to compare their data. Of the 204 IFA-seronegative participants in 2007, 36 (17.6%) were IFA seropositive in 2014; these participants presumably became infected after 2007. Of the 83 seropositive participants in 2007, 14 (16.9%) tested negative in 2014.

Analysis of the data collected from the MHS, microbiological laboratories, and the Herpen 2007 study revealed that 24.9% of the IFA-positive participants in 2014 (N = 128/513) previously tested positive. Although the laboratories informed the MHS of these 128 infections (because acute Q-fever is a notifiable disease), 78 (60.9%) of these cases did not meet the national notification criteria. Of the 513 positive participants in 2014, 51 (9.9%) had been notified previously by the MHS.

## Discussion

Seven years after a national Q-fever outbreak in the Netherlands, screening of 1,517 adults in one Dutch village revealed 33.8% seropositive participants and six participants with an IgG I titer ≥1:512. Two of these six participants were previously identified as having chronic Q-fever. Clinical evaluation of the remaining four individuals revealed one new chronic infection in a patient who had no prior history of acute Q-fever, no known cardiovascular risk factors, and no symptoms associated with an acute episode.

### Prevalence of Q-fever

To the best of our knowledge, this is the first large-scale seroprevalence study conducted in an entire village in order to identify patients with chronic Q-fever. The seroprevalence of antibodies against *C*. *burnetii* in our study (33.8%) is higher than the 12.2% reported among blood donors from high-incidence areas [5]. Lower and higher [11] IFA cut-off values are used for screening. Lacking an international standard we used the value commonly used in the Netherlands. Because the village population presumably was exposed to *C*. *burnetii* from 2007 through 2010, we expected to find evidence of waning immunity in 2014. Our finding that 16.9% participants seroreverted from IFA-positive to IFA-negative is consistent with a study in Wales that reported a serorevertion rate of 18% after six years [12]. Thus, our 2014 test results are likely an underestimation of the actual number of infections that occurred during the outbreak.

A recent study conducted among blood donors from high-incidence areas concluded that each notification might actually represent ≥12 infections [13]. In our study, 9.9% of IFA–positive participants were notified, confirming that the number of infections is approximately ten-fold greater than the number of notifications. Because these results were obtained from a village in which both the GPs and the general public are highly aware of Q-fever, we expect that even more infections went undiagnosed in other regions. Such underreporting is due primarily to asymptomatic infections, symptomatic but undiagnosed infections, and infections that do not meet our national notification criteria.

We found that 0.6% of seropositive participants in our study population developed chronic Q-fever, which is a lower rate than the 1.5–2% reported in the literature [5,6]. Serological testing within one year detects 98% [14] of the patients at risk for developing chronic Q-fever. However, the incubation time for chronic Q-fever is unknown and without further serological and clinical investigation can present years or even decades later [15].

### Strengths and limitations of the study

The primary strengths of our study are the large sample size, the high response rate (70.2% of the entire adult population in Herpen), the relative homogeneity of the study population, and the similarity between participants and non-participants. A limitation of our study is the possibility that individuals developed chronic Q fever earlier and died without being diagnosed. It cannot be excluded that those with severe disease were unable to participate, moved or also died since the outbreak. We have no information on non-participants and their Q fever status. We cannot exclude that one or more non-participants have chronic Q-fever. We cannot exclude potential bias caused by non-participation. Seropositive individuals could have been over represented if they desired to know their serological status because of certain risk factors. On the other hand they could have been under represented as they knew their long-term status due to serological follow-up. Those with an unknown Q-fever status or with a perceived risk for example occupational, might have shown increased interest in the study. Furthermore we could only contact those who were registered by the municipality but we expect the number of unregistered inhabitants to be very low.

## Conclusions

The Q-fever seroprevalence rate found in our study was remarkably high (34%), and 15% of the infected participants with at least one cardiovascular risk factor developed a chronic infection. Although our study revealed one new individual with chronic Q-fever, it is unlikely that screening other communities for chronic infections—particularly communities that were not as heavily exposed to *C*.*burnetii* during the outbreak—would yield significantly more infections. A policy of screening known high-risk groups for chronic infections in outbreak areas following an outbreak [8] might be more efficient and should be implemented rather than *ad hoc* population screening. A cost-effectiveness analysis should also be performed before initiating a population screening program for chronic Q-fever.

## Supporting Information

S1 Table IFA test results of 1517 participants. The six potential chronic cases are shown in bold italics. *Positive means a titer ≥1:64. The sample was however not titrated as phase I was not higher than 1:64. This made titration-in order to detect chronic Q-fever- unnecessary.
(DOC)

S1 Text Laboratory material and methods. (DOC)
