# Supplementary material for: Population Screening for Chronic Q-Fever Seven Years after a Major Outbreak
Source: PLoS One. 2015 Jul 1;10(7):e0131777. doi: 10.1371/journal.pone.0131777 (PMC4489093; doi:10.1371/journal.pone.0131777)
Supplement: S1 Table — The six potential chronic cases are shown in bold italics. *Positive means a titer ≥1:64. The sample was however not titrated as phase I was not higher than 1:64. This made titration-in order to detect chronic Q-fever- unnecessary. (DOC) [file pone.0131777.s001.doc]

**S1 Table. IFA test results of 1517 participants.**

The six potential chronic cases are shown in bold italics.

| **IgG Phase I** | **IgG Phase II** | N | **(%)** |
| --- | --- | --- | --- |
|  | **Negative test results** | **1004** | **(66.2)** |
| <1:64 | <1:64 | 1004 |  |
|  | **Positive test results** | **513** | **(33.8)** |
| <1:64 | 1:64 | 5 |  |
| <1:64 | positive* | 402 |  |
| <1:64 | 1:256 | 1 |  |
| 1:64 | <1:64 | 1 |  |
| 1:64 | 1:64 | 4 |  |
| 1:64 | positive | 13 |  |
| 1:64 | 1:128 | 2 |  |
| 1:64 | 1:256 | 8 |  |
| 1:64 | 1:512 | 9 |  |
| 1:64 | 1:1024 | 1 |  |
| 1:64 | >1:1024 | 2 |  |
| 1:128 | 1:128 | 4 |  |
| 1:128 | 1:256 | 5 |  |
| 1:128 | >1:256 | 1 |  |
| 1:128 | 1:512 | 11 |  |
| 1:128 | 1:1024 | 10 |  |
| 1:128 | >1:1024 | 10 |  |
| 1:256 | >1:256 | 3 |  |
| 1:256 | 1:256 | 2 |  |
| 1:256 | 1:512 | 3 |  |
| 1:256 | 1:1024 | 3 |  |
| 1:256 | >1:1024 | 4 |  |
| 1:256 | 1:2048 | 3 |  |
| ***1:512*** | ***1:4096*** | ***2*** |  |
| ***1:1024*** | ***1:1024*** | ***1*** |  |
| ***1:1024*** | ***1:2048*** | ***2*** |  |
| ***1:1024*** | ***1:4096*** | ***1*** |  |

*Positive means a titer ≥1:64. The sample was however not titrated as phase I was not higher than 1:64. This made titration -in order to detect chronic Q-fever- unnecessary.
