# Supplementary material for: Population Screening for Chronic Q-Fever Seven Years after a Major Outbreak
Source: PLoS One. 2015 Jul 1;10(7):e0131777. doi: 10.1371/journal.pone.0131777 (PMC4489093; doi:10.1371/journal.pone.0131777)
Supplement: S1 Text — (DOC) [file pone.0131777.s002.doc]

**S1 Text. Laboratory material and methods**

The IFA that we used for this study is a commercially available kit of Focus diagnostics, Cypress, CA, USA.

We used serum samples for the Q-fever polymerase chain reaction (PCR) test.

The gene that was targeted for the PCR test is the insertion element IS1111 of the *C. burnetii* genome of the Nine Mile strain. More information can be found in the article: Schneeberger, P. M., Hermans, M. H., van Hannen, E. J., Schellekens, J. J., Leenders, A. C., & Wever, P. C. (2010). Real-time PCR with serum samples is indispensable for early diagnosis of acute Q fever. Clinical and Vaccine Immunology, 17(2), 286-290. <http://cvi.asm.org/content/17/2/286.short>
